# Navigating the Volatile, Uncertain, Complex, and Ambiguous Landscape: Scripps Gerontology Center’s Path Forward

**DOI:** 10.1093/geroni/igaf122.1041

**Published:** 2025-12-31

**Authors:** Katherine Abbott

**Affiliations:** Miami University, Oxford, Ohio, United States

## Abstract

The Scripps Gerontology Center at Miami University, founded in 1922, has long been a leader in aging research. With a staff of 22 and 20 affiliated research fellows, Scripps operates in an increasingly volatile, uncertain, complex, and ambiguous (VUCA) environment. As 82% of our funding comes from external state and federal sources, we are grappling with the challenges of delayed grant reviews, uncertain indirect funding rates, and the need to support a graduate program while maintaining our commitment to innovation. In 2024, Scripps secured a $2.2 million investment from the University over five years to support the hiring of three researchers and a Director of Professional Education. This investment allows us to deepen our expertise in core areas while expanding into emerging fields. Additionally, relationships with philanthropic sources infused a $1.5 million dollar investment over three years to support our signature Opening Minds through Art program as well as a focus on broader dissemination of research findings. Operationally, Agile methodologies have enhanced efficiency and collaboration. However, the Department that awards master’s and doctoral degrees in gerontology faces challenges due to continued cuts to graduate assistant (GA) lines. While grant-funded GA positions remain an option, the higher costs associated with tuition waivers create financial constraints compared to hiring research staff directly. Despite these challenges, Scripps remains committed to our pillars of research, education and service. By navigating this VUCA landscape with strategic investments, operational innovation, and strong partnerships, we are positioning ourselves for continued excellence and impact in the years ahead.

